# Translation and Validation of the 5-Item Dry Eye Questionnaire into Lithuanian

**DOI:** 10.3390/medicina62101999

**Published:** 2026-10-15

**Authors:** Laura Mockaitienė, Almina Stramkauskaitė, Reda Žemaitienė

**Affiliations:** Department of Ophthalmology, Medical Academy, Lithuanian University of Health Sciences, LT-50161 Kaunas, Lithuania

**Keywords:** dry eye questionnaire, validity, reliability, dry eye syndrome, translation, Lithuanian

## Abstract

*Background and Objectives*: This study aimed to translate and validate the Lithuanian version of the 5-Item Dry Eye Questionnaire (DEQ-5). *Materials and Methods*: The original English DEQ-5 was translated and adapted into Lithuanian according to guidlines. A total of 40 Lithuanian-speaking adults without diagnosed ocular or systemic disease completed the questionnaire twice, with a 2–3-day interval. Internal consistency was evaluated using Cronbach’s alpha and corrected item–total correlations. Test–retest reliability was assessed using the intraclass correlation coefficient (ICC), and convergent validity was evaluated by correlating DEQ-5 scores with the Lithuanian version of the Ocular Surface Disease Index (OSDI). *Results*: The Lithuanian DEQ-5 demonstrated good internal consistency (Cronbach’s α = 0.843). Corrected item–total correlations ranged from 0.160 to 0.829, with Item 3 showing the weakest correlation. Test–retest reliability was good (ICC (A, 1) = 0.868; 95% CI: 0.764–0.928; *p* < 0.001). Bland–Altman analysis showed a mean difference (bias) of 0.075 points, with 95% limits of agreement ranging from −4.73 to 4.88 points. DEQ-5 scores showed a strong positive correlation with OSDI scores (Spearman’s ρ = 0.796; 95% CI: 0.55–0.92, *p* < 0.001), providing evidence of convergent validity in the OSDI subgroup. *Conclusions*: The Lithuanian version of the DEQ-5 demonstrated good internal consistency and test–retest reliability, while evidence of convergent validity was observed among participants who completed the OSDI.

## 1. Introduction

Dry eye disease (DED) is one of the most common ocular surface disorders, affecting an estimated 34.6% of the global population [1]. It occurs more frequently in women than in men, and its prevalence increases with age [2,3,4]. The development of DED is multifactorial and influenced by a combination of systemic and ocular diseases, hormonal changes, environmental factors, medication use, contact lens wear, refractive surgery, and nutritional deficiencies [5,6,7,8]. These factors can disrupt tear film homeostasis and damage the ocular surface, leading to symptoms such as ocular discomfort, dryness, irritation, fluctuating vision, and reduced visual performance [9,10]. Consequently, DED can substantially affect daily activities and quality of life [11]. Although there is currently no definitive cure, early diagnosis and appropriate management can effectively relieve symptoms, improve ocular surface health, and reduce the risk of disease progression. The severity and impact of the disease vary among individuals and depend on its underlying causes, symptom severity, and environmental influences. Beyond its impact on a person’s life, DED represents a substantial and growing public health and economic burden, reflecting both direct treatment costs and reduced work productivity [6].

Dry eye symptoms do not always correlate with objective clinical signs. Some patients report significant discomfort despite minimal clinical findings, while others have noticeable ocular surface changes but few symptoms [12].

The diagnosis of DED relies on both objective clinical tests and subjective patient-reported outcome measures. The most common clinical assessments include tear break-up time (TBUT), tear osmolarity, and ocular surface staining, whereas questionnaires capture the patient’s subjective experience [10,13,14]. The Tear Film & Ocular Surface Society Dry Eye Workshop II (TFOS DEWS II) Diagnostic Methodology report recommends the Ocular Surface Disease Index (OSDI) and the 5-item Dry Eye Questionnaire (DEQ-5) as validated instruments for standardized dry eye symptom assessment [15]. Although the OSDI is one of the most widely used questionnaires, its greater number of items and longer completion time may limit its practicality in routine clinical practice [10,16]. In contrast, the DEQ-5 is a brief questionnaire consisting of only five items that assess the frequency and intensity of ocular discomfort, dryness, and excessive tearing [17]. The DEQ-5 was derived by Chalmers et al. [17] from the longer Dry Eye Questionnaire (DEQ), which measures DED symptom frequency and diurnal intensity. The original DEQ is a self-administered questionnaire that assesses multiple DED symptoms including discomfort, dryness, and irritation by recording, for each symptom, how frequently it occurs and how intense it is in the morning and in the evening. By comparing morning and evening ratings, it captures the diurnal progression of symptoms that is characteristic of tear-deficient DED. The DEQ profiles each symptom individually, which is why the abbreviated five-item DEQ-5 was later derived from it for rapid screening [17]. The DEQ-5 questionnaire’s simplicity makes it a practical tool for rapid symptom assessment without imposing a significant burden on patients or clinicians [10,17].

Validated questionnaires such as the DEQ-5 promote a standardized approach to symptom evaluation, supporting both the diagnosis and follow-up of patients with DED. They also facilitate comparisons between studies by reducing variability associated with the use of different questionnaires and diagnostic criteria, which may partly explain the wide range of reported DED prevalence worldwide [10,17]. The DEQ-5 has been translated and validated in Chilean, Turkish, Portuguese, and Arabic populations. Across these adaptations, psychometric properties such as internal consistency, test–retest reliability, and validity have been evaluated [18,19,20,21]. However, linguistic equivalence alone does not guarantee that a questionnaire retains its psychometric properties across different cultural and linguistic settings, highlighting the importance of population-specific validation. To the best of our knowledge, the DEQ-5 has not yet been translated or validated for use in the Lithuanian population. A validated Lithuanian version would provide a standardized tool for evaluating dry eye symptoms in Lithuanian-speaking respondents and facilitate comparisons with international studies. Therefore, the aim of this study was to translate the DEQ-5 into Lithuanian and evaluate its psychometric properties in Lithuanian-speaking adults.

## 2. Materials and Methods

The study was conducted at the Hospital of Lithuanian University of Health Sciences (LUHS) Kaunas Clinics, Department of Ophthalmology after approval by the Research Ethics Committee (approval number: BE-2-77) and followed the Declaration of Helsinki. The DEQ-5 was utilized, comprising five questions, with two questions focusing on ocular discomfort, two on ocular dryness, and one on tearing. Questions 1a, 2a, and 3 offer five response options: 0—never, 1—rarely, 2—sometimes, 3—frequently, and 4—constantly. Questions 1b and 2b provide six options; the intensity of symptoms is rated on a scale from 0 to 5, with 0 indicating the absence of symptoms and 5 indicating very intense symptoms. The total DEQ-5 score ranges from 0 to 22, with higher scores reflecting greater symptom severity. Scores of 0–5 indicate minimal or no dry eye symptoms, scores of 6–11 are suggestive of a dry eye disease, and ≥12 may indicate the need for further evaluation to rule out Sjögren’s syndrome [15,17]. To translate and validate the original English version of the DEQ-5 into Lithuanian, the process proposed by Beaton et al. was followed [22]. The DEQ-5 is freely available for non-commercial research use, and the original authors were informed of the translation.

First, translation of the questionnaire into Lithuanian was performed by two translators—one by a professional translator with no medical background, and the other by a physician with C1-level English knowledge. The translated versions were then evaluated by a committee consisting of two ophthalmologists and an ophthalmology resident, resulting in a single Lithuanian version of the questionnaire. Subsequently, two independent back-translations from Lithuanian into English were performed by a professional translator and by a translator with basic knowledge of medical translation from English, both of whom were blinded to the original English version of the questionnaire and neither of whom had participated in the forward translation. The original English version and the back-translated versions were compared by the same committee. Then a pilot study was performed in a group of 10 senior ophthalmologists from the Department of Ophthalmology (7 women and 3 men; age range 40–60 years), none of whom participated in the subsequent test–retest study, in order to identify problems in comprehension of the questionnaire. After completing the questionnaire, each participant was asked whether the questions and the response options were understandable. All participants indicated that both were clear, and no changes to the wording were required; therefore, the final Lithuanian version of the DEQ-5 was established. The final version of the questionnaire was then administered to a sample of 40 individuals at two different times, separated by an interval of 2–3 days [22,23]. This sample size provided a respondent-to-item ratio of 8:1, which meets recommendations for cross-cultural adaptation and test–retest reliability studies [24]. Participants, including resident doctors, fellows, and nurses from the Department of Ophthalmology at LUHS, completed the questionnaires independently after providing informed consent. The study included Lithuanian-speaking adults aged 23–40 years without diagnosed ocular or systemic disease. Eligibility was established by a structured interview, and participants were excluded if they reported ocular or systemic disease, contact lens wear, use of ocular medications, previous ocular or refractive surgery, or use of systemic medications. Test–retest reliability requires that the underlying construct remains stable between administrations, which cannot be assumed in patients whose symptoms fluctuate or who receive treatment during the study interval. Selecting participants without diagnosed ocular or systemic disease minimized the influence of conditions or treatments that might affect dry eye symptoms.

No ophthalmological examination was performed, as the aim of the study was to evaluate the psychometric properties of a symptom-based questionnaire. Subclinical or undiagnosed dry eye could therefore not be excluded, which is consistent with the recognized discordance between dry eye symptoms and clinical signs [15]. The Lithuanian version of the OSDI was used to assess the convergent validity of the Lithuanian DEQ-5. Completion of the OSDI was optional during the second administration of the DEQ-5 questionnaire. The OSDI is a 12-item questionnaire assessing ocular symptoms, vision-related functioning, and environmental triggers over the preceding week [16]. Each item is scored from 0 to 4, and the total score is calculated on a scale from 0 to 100, with higher scores indicating greater symptom burden. Scores of 0–12 are considered within normal range, 13–22 indicate mild symptoms, 23–32 moderate symptoms, and 33–100 severe dry eye symptoms [16].

The analysis was conducted using the Statistical Package for Social Sciences (SPSS) version 31.0 (IBM Corporation, Armonk, NY, USA). The statistical analysis of the responses included calculating internal consistency using Cronbach’s alpha to assess the intercorrelation of questionnaire items, with a value ≥ 0.7 considered acceptable [25]. Test–retest reliability was evaluated using the intraclass correlation coefficient (ICC) with a two-way mixed-effects model and absolute agreement, single measures (ICC (A, 1)). ICC values below 0.50 were considered poor, 0.50–0.75 moderate, 0.75–0.90 good, and above 0.90 excellent [26]. Internal consistency was further evaluated using corrected item–total correlations. Correlation coefficients between 0.00 and 0.29 indicated weak associations, those between 0.30 and 0.49 indicated moderate associations, and values of 0.50 or higher reflected strong associations [27]. Agreement between test–retest DEQ-5 scores was additionally assessed using Bland–Altman analysis. The mean difference (bias) and 95% limits of agreement, calculated as the bias ± 1.96 SD of the differences, were reported [28]. To assess convergent validity, a subset of 21 participants completed both the Lithuanian DEQ-5 and the Lithuanian version of the OSDI, and Spearman’s rank correlation coefficient (ρ) was calculated to evaluate the correlation between the two questionnaires. Statistical significance was set at *p* < 0.05.

## 3. Results

A total of 40 participants completed the Lithuanian DEQ-5 questionnaire. The mean age was 29.02 ± 5.34 years, and the study population consisted of 10 men (25%) and 30 women (75%). No major difficulties were encountered during the translation or administration of the questionnaire.

The mean DEQ-5 score at the first administration was 7.28 ± 4.61 (range 0–18). Of the 40 participants, 17 (42.5%) scored 0–5, 15 (37.5%) scored 6–11, and 8 (20.0%) scored ≥12. Responses to Item 3 were: never, *n* = 15 (37.5%); rarely, *n* = 13 (32.5%); sometimes, *n* = 7 (17.5%); frequently, *n* = 4 (10.0%); constantly, *n* = 1 (2.5%). The Lithuanian version of the DEQ-5 demonstrated good internal consistency, with a Cronbach’s alpha coefficient of 0.843. Corrected item–total correlation coefficients ranged from 0.160 to 0.829. Four items showed strong correlations with the total score, whereas Item 3 demonstrated a weaker association (Table 1). Cronbach’s alpha increased from 0.843 to 0.922 when Item 3 was removed.

The test–retest reliability of the Lithuanian DEQ-5 was good, with an ICC (A, 1) of 0.868 (95% CI: 0.764–0.928, *p* < 0.001). Bland–Altman analysis showed a mean difference (bias) of 0.075 points (95% CI: −0.71 to 0.86), with 95% limits of agreement ranging from –4.73 to 4.88 points.

The OSDI questionnaire was completed by 21 participants, with a mean score of 11.51 ± 8.95 (range 0–29). Participants who completed the OSDI (*n* = 21) and those who did not (*n* = 19) did not differ significantly in the first administration DEQ-5 score (6.43 ± 4.41 vs. 8.21 ± 4.76; t = −1.229, *p* = 0.226). Among these participants, the Lithuanian DEQ-5 demonstrated a strong positive correlation with the OSDI (ρ = 0.796, 95% CI: 0.55–0.92, *p* < 0.001), providing evidence of the convergent validity of the translated questionnaire.

## 4. Discussion

The present study translated the DEQ-5 into Lithuanian and evaluated its psychometric properties in Lithuanian-speaking adults. The translation followed the internationally recognized methodology proposed by Beaton et al., ensuring conceptual and linguistic equivalence between the original and Lithuanian versions [22]. The adaptation process was completed without major linguistic challenges, indicating that the original concepts of the questionnaire were successfully preserved while ensuring its suitability for the Lithuanian-speaking population. Furthermore, the Lithuanian version demonstrated good psychometric properties, with good internal consistency and test–retest reliability, while evidence of convergent validity was observed among participants who completed the OSDI. Together, these findings support the reliability and convergent validity of the Lithuanian DEQ-5 in the population studied.

The Lithuanian version of the DEQ-5 demonstrated good internal consistency, with a Cronbach’s alpha coefficient of 0.843. This finding is comparable to those reported for the Chilean (α = 0.8085), Arabic (α = 0.878), Portuguese (α = 0.887), and Turkish (α = 0.90) versions of the questionnaire, indicating that the DEQ-5 maintains good internal consistency across different language adaptations [18,19,20,21].

Item 3 showed a markedly weaker corrected item–total correlation (0.160) than the remaining four items (0.754–0.829), and its removal increased Cronbach’s alpha from 0.843 to 0.922. This may reflect a conceptual rather than a translational issue, as Items 1a, 1b, 2a, and 2b assess ocular discomfort and dryness, whereas Item 3 assesses watery eyes, which may result from reflex tearing in DED. A similar pattern was observed in the Arabic version, where removal of Item 3 increased Cronbach’s alpha from 0.878 to 0.924 [21], suggesting that this finding may reflect the structure of the instrument rather than the Lithuanian translation. The variability of Item 3 responses (SD 1.10) was comparable to that of the other items (SD 1.08–1.30), indicating that its weak item–total correlation was not due to restricted variance. As cross-cultural adaptation aims to preserve conceptual equivalence rather than maximize internal consistency, and excessive tearing is a clinically meaningful manifestation of DED that contributes to the discriminative performance of the DEQ-5 [17], Item 3 was retained despite its limited contribution to internal consistency in this sample.

The mean DEQ-5 score in our study (7.28 ± 4.61) was lower than that reported in the Arabic validation study (9.0 ± 5.2) [21]. Although both studies included adults without diagnosed ocular or systemic disease, this difference may reflect variations in the characteristics of the study populations, as well as differences in environmental and lifestyle factors that may influence dry eye symptoms [29,30]. More than half of our participants (57.5%) scored ≥6, the threshold suggestive of DED. Participants were defined as healthy based on the absence of diagnosed ocular or systemic disease, and symptomatic but undiagnosed dry eye was therefore not excluded. Dry eye symptoms are common among young adults, particularly with prolonged digital device use, and symptoms correlate poorly with clinical signs [12,31]. The DEQ-5 cut-off values are screening thresholds rather than diagnostic criteria and were derived from specific study populations [15,17]. This distribution of scores was nevertheless advantageous for the reliability analyses, as it provided sufficient variability.

The good test–retest reliability observed in the Lithuanian version (ICC = 0.868; 95% CI: 0.764–0.928; *p* < 0.001) is comparable to that reported for the Arabic (ICC = 0.887; 95% CI: 0.819–0.931; *p* < 0.001) and Turkish (ICC = 0.95; 95% CI: 0.90–0.98; *p* < 0.001) versions. Although the Turkish version demonstrated a slightly higher ICC, all three studies indicate good to excellent test–retest reliability, supporting the reproducibility of the DEQ-5 across different language adaptations [19,21]. Bland–Altman analysis showed minimal systematic bias between test and retest scores (0.075 points), supporting good overall agreement between the two measurements, with 95% limits of agreement ranging from −4.73 to 4.88 points.

The strong positive correlation observed between the Lithuanian DEQ-5 and the OSDI provides evidence of the convergent validity of the translated questionnaire. These findings agree with those reported by Akowuah et al., who demonstrated that the DEQ-5 is a valid instrument for assessing dry eye symptoms in both clinical and epidemiological settings [32]. Although both questionnaires evaluate subjective symptoms of dry eye disease, the OSDI primarily focuses on symptom frequency and their impact on daily activities, whereas the DEQ-5 additionally assesses symptom intensity [16,17]. Despite its brevity, the DEQ-5 captures the key symptoms of dry eye disease, including ocular discomfort, dryness, and excessive tearing, and has been shown to distinguish between different levels of disease severity, supporting its role as a practical screening tool in clinical settings [17,32]. These characteristics make the DEQ-5 a practical instrument for symptom assessment, particularly where consultation time is limited. The availability of a Lithuanian version enables standardized symptom assessment in Lithuanian-speaking respondents and direct comparison of Lithuanian data with international studies, although its performance in patients with dry eye disease has not yet been evaluated.

This study has several limitations. Although the sample size was relatively small, it exceeded the recommended minimum respondent-to-item ratio of 5:1 [24] and was comparable to sample sizes used for test–retest reliability assessment in previous cross-cultural validation studies of the DEQ-5 [17,18]. Nevertheless, larger samples would provide greater precision. Convergent validity was assessed in a subset of 21 participants because completion of the OSDI was optional. The observed correlation was strong; however, given the small sample size, this estimate should be interpreted with caution. Participants were young adults recruited from a single ophthalmology department, and their professional familiarity with ocular symptoms may limit the generalizability of the findings to the broader Lithuanian population. A similarly selected sample was used in the Portuguese validation study, which included residents, fellows, assistant physicians, and nursing staff from a single ophthalmology department [20]. The sample comprised predominantly women (75%). As dry eye symptoms are more prevalent in women, this sex imbalance should be considered when generalizing the present findings. Health status was determined by a structured interview rather than an ophthalmological examination; therefore, subclinical or undiagnosed dry eye could not be excluded, and the DEQ-5 cut-off values could not be clinically verified. Finally, consistent with published validations of other language versions of the DEQ-5, the present study focused on internal consistency, test–retest reliability, and convergent validity, while known-groups validity, diagnostic accuracy, and responsiveness to treatment were not assessed.

Future studies should evaluate the known-groups validity of the Lithuanian DEQ-5 by comparing patients with clinically diagnosed DED and healthy controls. Longitudinal studies are needed to assess responsiveness to treatment and the potential usefulness of the questionnaire for monitoring symptoms after cataract or refractive surgery. These investigations would help establish the clinical applicability of the Lithuanian DEQ-5 in different patient populations.

## 5. Conclusions

In conclusion, the Lithuanian version of the DEQ-5 demonstrated good internal consistency and test–retest reliability, while evidence of convergent validity was observed among participants who completed the OSDI. Its use may facilitate symptom assessment and future clinical and epidemiological research in Lithuania.

## Figures and Tables

**Table 1:** Corrected item–total correlations and Cronbach’s alpha if item deleted for the Lithuanian version of the DEQ-5.

Question	Mean ± SD	Corrected Item-Total Correlation	Cronbach’s Alpha If Item Deleted
1a	1.75 ± 1.08	0.829	0.766
1b	1.60 ± 1.28	0.811	0.763
2a	1.43 ± 1.11	0.774	0.779
2b	1.43 ± 1.30	0.754	0.780
3	1.07 ± 1.10	0.160	0.922

## Data Availability

The original contributions presented in this study are included in the article. Further inquiries can be directed to the corresponding author.

## Abbreviations

The following abbreviations are used in this manuscript:

TFOS DEWS	The Tear Film & Ocular Surface Society Dry Eye Workshop II
DEQ-5	5-Item Dry Eye Questionnaire
ICC	Intraclass correlation coefficient
OSDI	Ocular Surface Disease Index
DED	Dry eye disease
TBUT	Tear break-up time
LUHS	Lithuanian University of Health Sciences
SPSS	Statistical Package for Social Sciences
